# Is regular in-person recall superior to non-regular in-person recall in clinical outcomes among new patients undergoing peritoneal dialysis

**DOI:** 10.1080/0886022X.2022.2145972

**Published:** 2022-11-21

**Authors:** Ying Liu, Wen Xiu Chang, Ben-Chung Cheng, Jin-Bor Chen

**Affiliations:** a Department of Nephrology, Tianjin First Center Hospital, Tianjin, China; b Division of Nephrology, Department of Internal Medicine, Kaohsiung Chang Gung Memorial Hospital and School of Medicine, College of Medicine, Chang Gung University, Taiwan, Republic of China

**Keywords:** Adherence, clinical outcomes, hospitalization, in-person recall, peritoneal dialysis, peritonitis

## Abstract

**Objective:**

To investigate the different impacts on clinical outcomes between regular recall and non-regular recall among incident peritoneal dialysis (PD) patients.

**Methods:**

A two-center cohort of 216 new PD patients from 1January 2013, to 31 December 2014, was studied. Informative clinical data were collected from baseline until two years after PD initiation, including demographics, laboratory and PD-related parameters, PD-related peritonitis rates, and frequency of hospitalization. Regular in-person recall (RPR) was defined as having a one-month interval and non-regular in-person recall (NRPR) as an interval ranging from more than one month to less than three months.

**Results:**

Percentage of patients with peritonitis was significantly higher among patients in the NRPR group than among those in the RPR group (27.7% vs. 16.5%, *p* = .049). PD-related peritonitis rate was higher in the NRPR vs. RPR cohorts (0.16 vs. 0.09 person/year, *p* = .019). PD-related hospitalization frequency was also higher in the NRPR cohort (0.8 ± 1.0 vs. 0.5 ± 0.9, *p* = .039) over two years. Kt/*V* values in the NRPR cohort gradually decreased over two years and were at lower levels than in the RPR cohort.

**Conclusions:**

New PD patients with NRPR showed higher rates of PD-related peritonitis and hospitalization frequency than patients with RPR.

## Introduction

The incidence of end-stage renal disease is increasing annually, and it is emerging as a critical health issue. Peritoneal dialysis (PD) is one of the two main modalities for renal replacement therapy in end-stage renal disease and is used by approximately 11% of the dialysis population worldwide [1]. PD offers home treatment and requires daily compliance and high involvement of patients and/or caregivers to guarantee favorable outcomes. To some extent, the choice to use PD is determined by patients’ subjective wishes. Adherence to treatment is critical because studies have shown that noncompliance to treatment can lead to increased costs for medical care [2–4]. In PD studies, noncompliance to treatment also has serious consequences including increased risk of mortality and hospitalization [5,6].

Prior investigations on noncompliance to treatment have commonly focused on the hemodialysis population. Noncompliance in PD patients has been less reported. Studies on compliance of PD patients are based on three aspects of treatment: noncompliance with dialysis procedures/exchanges, medications, and diet/fluid restrictions [5–13]. The majority of studies focused on adherence to dialysis and drug treatment, with adherence to dialysis exchange evaluated most commonly [14].

Although some variables were identified as affecting noncompliance, there was almost no consensus among the observed association studies [14]. In one study, poor compliance with routine interval visits to nephrologists is a risk factor for all-cause mortality in remote PD patients. Therefore, visits to nephrologists at regular intervals in PD centers or satellite PD centers should be encouraged for better clinical outcomes by investigators’ recommendations [15]. However, there are no studies evaluating compliance to regular outpatient recall in the PD population, or its impact on clinical outcomes. This study was conducted to investigate the impact on clinical outcomes between regular in-person recall (RPR) and non-regular in-person recall (NRPR) among incident PD patients.

## Materials and methods

### Patient selection

Study design was a retrospective medical information review over two-year study period. New patients diagnosed with end-stage renal disease who initiated PD therapy in an outpatient clinic and no prior hemodialysis were selected for analysis. Patients were recruited from two hospital-facilitated PD centers, Tianjin First Center Hospital in China and Kaohsiung Chang Gung Memorial Hospital in Taiwan, from 1 January 2013, to 31 December 2014. All participants were followed up in the outpatient clinic of their respective hospitals for two years until PD termination, transfer to hemodialysis, receiving kidney transplantation or death.

### Data collection

Analytic information included demographics, laboratory, and PD-related parameters and clinical outcomes. Laboratory data and the parameters of standard peritoneal equilibration tests (PET) were generally collected at 1, 12, and 24 months after PD initiation. Data on PD-related parameters were collected from PD home records and PET, including urine output, dialysis adequacy, residual renal function estimated by creatinine clearance, and glucose exposure amount in PD solution dwell. All hemograms and biochemical samples were measured using commercial kits and an autoanalyzer. Albumin levels were measured using the bromocresol green method. Clinical outcomes included drop outs, PD-related peritonitis, and hospitalization. PD-related hospitalizations were defined as PD-related peritonitis, catheter-related infection, catheter displacement, inadequate PD, and fluid overload. PD dropout was defined by PD termination caused by death, ultrafiltration failure, failed PD-related peritonitis treatment, or hemodialysis shifting. Technique failure was defined by PD termination caused by ultrafiltration failure, treatment failure for PD-related peritonitis, or hemodialysis shifting.

### Care pathway in PD patients

The content of follow-up care pathway in PD patients is followed by International Society of PD (ISPD) guidelines [16] in study hospitals. Briefly, The program consisted of face-to-face counseling by nephrologists and PD nurses, telephone call counseling by PD nurses, home visit when necessary and 24-h hotline for urgent circumstances. The informative content of standard operative procedures in PD outpatient clinics were shown in Online Resource 1 (Supplemental Table S1).

### Definition of in-person recall in outpatient clinic

In practice, PD patients were recalled to PD units at one-month intervals in study hospitals. RPR in an outpatient clinic was defined as a visit involving face-to-face interviews between nephrologists/PD nurses and patients at one-month intervals and NRPR as visits at intervals ranging from more than one month to less than three months. Non-adherence to outpatient recall was defined when patients were unwilling to abide by the rule of one-month interval recall.

### Statistical analysis

The baseline characteristics and laboratory measurements of the participants are summarized and presented as mean ± standard deviation (SD), median (interquartile range), and frequency (percentage) (Tables 1 and 2). Differences in baseline characteristics and clinical outcomes between the groups were estimated by using independent two-sample *t*-test, chi-squared test, binomial probability test, or Fisher’s exact test (Table 1, Supplemental Tables S5, S6). The changes of baseline laboratory measurements within the two groups were estimated using one-way repeated measures ANOVA (Table 2). Differences in laboratory measurements and PET characteristics at baseline and over two-year period between groups were estimated by using independent two-sample *t*-test and one-way repeated measures ANOVA (Table 3 and Supplementary Tables S4, S8). The association between the PET-related parameters and visiting frequency in the study period was analyzed using an independent two-sample *t*-test to determine the difference between the groups (Table 4). The values *p*^a^ and *p*^b^ were estimated using one-way repeated measures ANOVA to determine the changes in PET-related parameters between groups and within groups, respectively (Table 4). The value of *p*^c^ was estimated using repeated measures ANOVA to determine the time-varying changes between groups (Table 4).

**Table 1.** Demography at baseline and clinical outcomes.

Variables	Total	Non-regular recall	Regular recall	*p*
Patients	216	83	133	
Age (y)	52.5 ± 14.0	50.3 ± 14.7	53.9 ± 13.4	.067
BMI(kg/m^2^)	23.5 ± 4.0	23.2 ± 4.1	23.8 ± 3.9	.296
Sex				.420
Male	107 (49.5%)	44 (53.0%)	63 (47.4%)	
Female	109 (50.5%)	39 (47.0%)	70 (52.6%)	
Marital status				.091
Married	184 (85.2%)	75 (90.4%)	109 (82.0%)	
Others	32 (14.8%)	8 (9.6%)	24 (18.0%)	
Education				.139
Bachelor	45 (20.8%)	13 (15.7%)	32 (24.1%)	
Others	171 (79.2%)	70 (84.3%)	101 (75.9%)	
Employment status				.156
Unemployed	163 (75.5%)	67 (80.7%)	96 (72.2%)	
Employed	53 (24.5%)	16 (19.3%)	37 (27.8%)	
Caregiver				.302
No	179 (82.9%)	66 (79.5%)	113 (85%)	
Yes	37 (17.1%)	17 (20.5%)	20 (15%)	
Smoking	28 (13.0%)	19 (22.9%)	9 (6.8%)	.001
Alcohol drinking	20 (9.3%)	11 (13.3%)	9 (6.8%)	.110
Etiology of CKD				.470
Non-DM	158 (73.1%)	63 (75.9%)	95 (71.4%)	
DM	58 (26.9%)	20 (24.1%)	38 (28.6%)	
Comorbidities				
Hypertension	157 (72.7%)	81 (97.6%)	76 (57.1%)	<.001
CAD	69 (31.9%)	44 (53.0%)	25 (18.8%)	<.001
DM	56 (25.9%)	19 (22.9%)	37 (27.8%)	.421
Cerebral stroke	13 (6.0%)	11 (13.3%)	2 (1.5%)	.001
Hepatitis B/C	4 (1.9%)	2 (2.4%)	2 (1.5%)	.639
Clinical outcomes				
Drop-out(*n*,%)	31 (14.4%)	9 (10.8%)	22 (16.5%)	.245
Peritonitis(*n*,%)	45 (20.8%)	23 (27.7%)	22 (16.5%)	.049
Peritonitis rate (per patient-year)	0.11	0.16	0.09	.019
Peritonitis frequency	0.3 ± 0.7	0.4 ± 0.6	0.2 ± 0.7	.237
Hospitalization	131 (60.6%)	53 (63.9%)	78 (58.6%)	.446
Hospitalization frequency (all causes)	1.5 ± 1.8	1.5 ± 1.5	1.6 ± 1.9	.744
Hospitalization frequency (PD causes)	0.6 ± 0.9	0.8 ± 1.0	0.5 ± 0.9	.039

BMI: body mass index; CKD: chronic kidney disease; DM: diabetes mellitus; CAD: coronary artery disease; PD: peritoneal dialysis.

**Table 2.** Laboratory measurements at baseline and over two-year period.

Variables	Baseline	12-month	24-month	*p*
All patients				
Hb (g/dL)	10.8 ± 1.7	10.7 ± 1.6	10.6 ± 1.5	.049
Alb (g/dL)	3.7 ± 0.5	3.8 ± 0.4	3.7 ± 0.4	<.001
BUN (mg/dL)	59.8 (46.6–70.2)	61.0 (51.2–73.0)	57.0 (48.9–71.0)	.129
Cr (mg/dL)	9.7 ± 3.1	10.8 ± 2.9	11.2 ± 3.1	<.001
Ca (mg/dL)	8.9 ± 1.0	9.2 ± 0.9	9.2 ± 0.8	<.001
P (mg/dL)	5.0 ± 1.2	5.4 ± 1.3	5.5 ± 1.5	<.001
Na (mEq/L)	139.0 ± 4.7	138.4 ± 5.0	137.8 ± 5.6	.001
K (mEq/L)	4.2 ± 0.8	4.3 ± 0.7	4.2 ± 0.8	.369
iPTH (pg/mL)	202.9 (102.1–374.6)	253.5 (112.2–377.0)	278.2 (119.2–424.0)	.027
Non-regular recall				
Hb (g/dL)	11.4 ± 1.9	11.2 ± 1.6	11.0 ± 1.6	.068
Alb (g/dL)	3.4 ± 0.5	3.7 ± 0.5	3.6 ± 0.5	<.001
BUN (mg/dL)	53.8 (42.1–64.3)	56.1 (47.7–67.9)	53.0 (42.6–65.0)	.754
Cr (mg/dL)	8.4 ± 2.3	9.8 ± 2.9	10.4 ± 3.2	<.001
Ca (mg/dL)	8.5 ± 1.0	8.8 ± 1.1	8.8 ± 0.7	.004
P (mg/dL)	5.0 ± 1.2	5.3 ± 1.4	5.2 ± 1.7	.206
Na (mEq/L)	141.8 ± 3.8	142.6 ± 2.9	142.0 ± 3.8	.235
K (mEq/L)	4.5 ± 0.8	4.5 ± 0.7	4.3 ± 0.8	.108
iPTH (pg/mL)	192.4 (113.8–315.9)	268.8 (138.4–352.7)	236.8 (111.7–410.8)	.125
Regular recall				
Hb (g/dL)	10.5 ± 1.6	10.4 ± 1.5	10.3 ± 1.3	.459
Alb (g/dL)	3.8 ± 0.4	3.8 ± 0.4	3.8 ± 0.4	.211
BUN (mg/dL)	64.0 (51.0–77.0)	64.0 (55.5–75.0)	61.5 (52.5–73.5)	.124
Cr (mg/dL)	10.5 ± 3.2	11.5 ± 2.8	11.6 ± 3.0	<.001
Ca (mg/dL)	9.2 ± 1.0	9.4 ± 0.8	9.4 ± 0.8	<.001
P (mg/dL)	5.1 ± 1.3	5.4 ± 1.2	5.8 ± 1.4	<.001
Na (mEq/L)	137.2 ± 4.3	135.9 ± 4.3	135.2 ± 5.0	<.001
K (mEq/L)	4.1 ± 0.8	4.1 ± 0.6	4.1 ± 0.7	.731
iPTH (pg/mL)	226.0 (99.8–506.0)	242.4 (100.2–383.4)	307.2 (135.8–493.5)	.133

Abbreviations: Hb: hemoglobin; Alb, albumin; BUN: blood urea nitrogen; Cr, creatinine; Ca: calcium; P: phosphate; Na: sodium; K: potassium; iPTH: intact parathyroid hormone.

**Table 3.** Comparative results of laboratory measurements in study population over time.

Variables	All ^a^ (over time)	NRPR ^a^ (over time)	RPR ^a^ (over time)	NRPR vs RPR ^b^ (Baseline)	NRPR vs RPR ^b^ (12-month)	NRPR vs RPR ^b^ (24-month)	NRPR vs RPR ^c^ (over time)
Hb (g/dL)	0.049	0.068	0.459	<0.001	<0.001	0.002	0.795
Alb (g/dL)	<0.001	<0.001	0.211	<0.001	0.065	0.002	0.042
BUN (mg/dL)	0.129	0.754	0.124	0.001	<0.001	0.001	0.959
Cr (mg/dL)	<0.001	<0.001	<0.001	<0.001	<0.001	0.008	0.336
Ca (mg/dL)	<0.001	0.004	<0.001	<0.001	<0.001	<0.001	0.790
P (mg/dL)	<0.001	0.206	<0.001	0.749	0.536	0.009	0.112
Na (mEq/L)	0.001	0.235	<0.001	<0.001	<0.001	<0.001	0.010
K (mEq/L)	0.369	0.108	0.731	<0.001	<0.001	0.098	0.227
iPTH (pg/mL)	0.027	0.125	0.133	0.047	0.287	0.191	0.806

NRPR: non-regular in-person recall; RPR: Regular in-person recall; Hb: hemoglobin; Alb, albumin; BUN: blood urea nitrogen; Cr, creatinine; Ca: calcium; P: phosphate; Na: sodium; K: potassium; iPTH: intact parathyroid hormone.

^a^*p*-Value were estimated using one-way repeated measures ANOVA to determine the changes in laboratory measurements over time within groups.

^b^*p*-Value were estimated using independent two-sample *t*-test to determine the changes in laboratory measurements at single time point between groups.

*^c^p*-Value were estimated using two-way repeated measures ANOVA to determine the changes in laboratory measurements between groups over times.

**Table 4.** The repeated measurements of PD-related parameters over two-year period.

Variables	Total	Non-regular recall	Regular recall	*p^a^*
Glucose exposure (gm/Day)				
Baseline	112.5 (90.0–152.0)	90.0 (90.0–120.0)	130.0 (97.5–187.5)	<.001
12-month	121.3 (90.0–163.0)	120.0 (90.0–160.0)	130.5 (97.5–187.5)	.015
24-month	140.0 (97.5–181.0)	140.0 (110.0–180.0)	130.5 (97.5–187.5)	.864
*p^b^*	<.001	<.001	.254	
*p^c^*		<.001		
Urine amount (L/Day)				
Baseline	0.9 ± 0.6	1.0 ± 0.6	0.9 ± 0.6	.047
12-month	0.7 ± 0.6	0.8 ± 0.6	0.7 ± 0.6	.192
24-month	0.5 ± 0.5	0.5 ± 0.5	0.4 ± 0.5	.388
*p^b^*	<.001	<.001	<.001	
*p^c^*		.345		
Kt/*V* (total)				
Baseline	2.1 ± 0.5	2.0 ± 0.5	2.1 ± 0.5	.195
12-month	2.0 ± 0.5	1.9 ± 0.5	2.1 ± 0.4	.001
24-month	2.0 ± 0.4	1.8 ± 0.5	2.0 ± 0.4	<.001
*p^b^*	<.001	<.001	.226	
*p^c^*		.023		
Renal Ccr(weekly) (L/w/1.73m^2^)				
Baseline	7.2 (3.8–25.8)	32.9 (19.3–46.8)	4.3 (3.2–5.9)	<.001
12-month	5.1 (3.4–16.2)	18.9 (6.8–33.7)	4.3 (3.2–5.9)	<.001
24-month	4.7 (2.7–9.5)	8.4 (0–19.6)	4.1 (2.9–5.7)	<.001
*p^b^*	<.001	<.001	.024	
*p^c^*		<.001		
Total Ccr (weekly) (L/w/1.73m^2^)				
Baseline	63.8 (53.8–83.6)	60.2 (49.3–71.3)	65.3 (56.2–87.5)	.008
12-month	58.7 (51.2–70.6)	53.1 (45.3–64.3)	61.7 (53.9–73.6)	.059
24-month	56.1 (47.8–65.7)	50.6 (40.6–62.0)	58.0 (52.3–67.7)	.232
*p^b^*	<.001	.023	<.001	
*p^c^*		.389		

Ccr: creatinine clearance.

*p*^a^ were estimated using independent two-sample *t*-test and *p*^b^ were estimated using one-way repeated measures ANOVA to determine the changes in PET-related parameters between groups and within groups, respectively.

*p^c^* were estimated using two-way repeated measures ANOVA to determine the difference of PET-related parameters between groups over times, respective.

**Figure 1.**
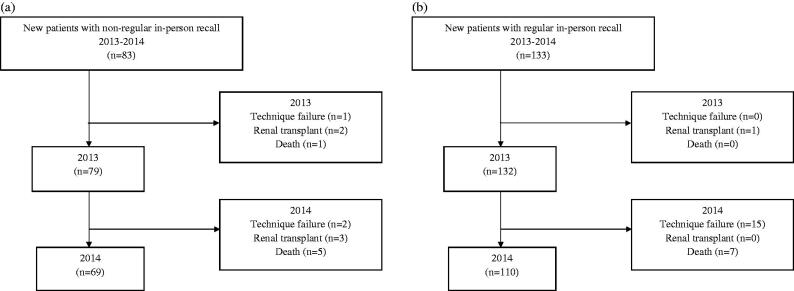
Summary of clinical outcomes of patients in the (a) non-regular in-person recall group and (b) regular in-person recall group.

Cox proportional hazard regression with time-varying covariates were used to derive adjusted hazard ratios (HR) for peritonitis (Table 5). Comparisons between times to recall and clinical outcomes were estimated by using independent two-sample *t*-test (Supplementary Table S2). PD mode changes in patients with incremental PD were estimated by using McNemar’s test (Supplementary Table S3).The clinical outcomes in participants were summarized and presented as frequency (percentage) (Figure1). The 95% confidence interval (CI) and *p*-value were used to determine statistical significance. A *p* value less than 0.05 was considered statistically significant. All statistical analyses were conducted using STATable TA version 11.1 (Stata Corp. 2009. Stata Statistical software: Release 11. College Station, Texas, Stata Corp LP., USA).

**Table 5.** Cox proportional hazard regression with time-varying covariates for peritonitis.

Variables	Univariate		Full-adjusted model	
	HR (95% CI)	*p*	HR (95% CI)	*p*
Regular visit	0.56 (0.31–1.01)	.055	0.59 (0.38–0.91)	.017
Age(y)	1.02 (0.99–1.04)	.133	1.02 (1.003–1.03)	.015
Sex, male	0.62 (0.34–1.12)	.111	0.57 (0.37–0.87)	.008
Marital status, married	0.54 (0.19–1.50)	.233	0.88 (0.45–1.70)	.695
Education, bachelor	1.01 (0.49–2.10)	.972	0.70 (0.44–1.11)	.132
CKD cause, DM	0.57 (0.26–1.22)	.145	0.26 (0.12–0.56)	<.001
Comorbidities, DM	0.81 (0.40–1.63)	.546	1.63 (0.80–3.31)	.175
Time-varying covariates				
APD vs CAPD	1.09 (0.83–1.43)	.538	0.83 (0.61–1.12)	.215
Hb (g/dL)	1.08 (0.99–1.18)	.066	1.1 (1.003–1.21)	.044
Alb (g/dL)	0.54 (0.41–0.71)	<.001	0.47 (0.35–0.64)	<.001
K (mEq/L)	0.95 (0.79–1.14)	.599	1.005 (0.83–1.22)	.963
iPTH (pg/mL)	1.0001 (0.99–1.001)	.809	1.0004 (0.9999–1.0009)	.118
Ferritin (ng/mL)	0.99999 (0.9998–1.0002)	.989	0.99999 (0.9998–1.0002)	.910
Glucose exposure (gm/Day)	1.001 (0.99–1.004)	.317	1.003 (0.9997–1.01)	.081
Urine amount (L/Day)	0.98 (0.79–1.22)	.860	1.01 (0.78–1.32)	.930
Kt/*V* (total)	0.80 (0.58–1.12)	.190	1.04 (0.68–1.59)	.858
Total Ccr (L/w/1.73m^2^)	0.999 (0.995–1.01)	.969	1.0004 (0.99–1.01)	.915

HR: hazard ratio.

CI: confidence interval.

## Results

A total of 216 new patients underwent PD were identified for analysis, with 133 patients in the RPR and 83 patients in the NRPR group. Visit interval was one month in the RPR group, and mean visit interval was 1.45 ± 0.35 months (range, 1–2.59 months) in the NRPR group. The mean age was 53.9 years in the RPR group and 50.3 years in the NRPR group. Sex showed equal distribution in the two groups. The causes of NRPR in the outpatient clinic were personal events on follow-up days (*n* = 28), non-adherence for personal preference (*n* = 22), distance from the hospital (*n* = 17), disease episodes on follow-up days (*n* = 13), and financial reasons for traffic transportation (*n* = 3). Among social features, a higher percentage of smoking was identified in patients with NRPR than in those with RPR (*p* = .001). Other features including age, sex, body mass index, education, status of employment, and caregiver/marital status were not significantly different between the two groups. Patients in the NRPR group showed a higher percentage of comorbidities with hypertension, coronary artery disease, and cerebral stroke compared with those in the RPR group. Among clinical outcomes, percentage of patients with PD-related peritonitis was significantly higher in the NRPR group than in patients in the RPR group (27.7% vs. 16.5%, *p* = .049), whereas percentage of drop outs and all-cause hospitalization was not higher in the NRPR group. PD-related peritonitis rate was higher in the NRPR group than the RPR group (0.16 vs. 0.09 person-year, *p* = .019). Frequency of PD-related hospitalizations was significantly higher in the NRPR group than in the RPR group (0.8 ± 1.0 vs. 0.5 ± 0.9, *p* = .039), whereas not significant in all-cause hospitalizations over two years (Table 1). Detailed information about missed recall appointments in outpatient clinics and their contribution to clinical outcomes and PD mode changes in patients with incremental PD therapy are shown in Online Resource 2 (Supplemental Table S2) and Online Resource 3 (Supplemental Table S3), respectively. There were no significant differences in rates of drop out, PD-related peritonitis, and hospitalization based on stratification of missed times of recall in outpatient clinic over the study period.

Serial laboratory data over two years and comparison between RPR group and NRPR group are shown in Tables 2 and 3. For albumin measurements, the RPR cohort showed more stable serum albumin levels over two years, whereas the NRPR cohort showed temporary changes in serum albumin levels during the study period (*p* < .001). For blood urea nitrogen measurements, the NRPR cohort showed temporary changes in blood levels, whereas the RPR cohort showed a decreasing tendency in blood levels (*p* = .124). Serum creatinine levels showed significant increases in both groups over two years (*p* < .001). Similar changes were also observed in serum calcium and phosphate levels in the RPR group (*p* < .001).

Regarding PD modes, more patients in the NRPR group used automated PD at baseline than those in the RPR (80.7% vs. 43.6%, *p* < 0001) group with tendency through two years. The detailed information in PET are shown in Online Resource 4 (Supplemental Table S4). Glucose exposure showed an increasing trend over two years in patients in the NRPR group, whereas patients in the RPR group maintained a relatively constant exposure amount. Dialysis adequacy index (Kt/*V*) showed a decreasing trend in the NRPR group but not in the RPR group. Daily urine output and total weekly creatinine clearance showed significantly decreasing trends in both cohorts, as shown in Table 4.

In analyzing the risk for peritonitis over two years by fully adjusted model, RPR (HR 0.59, 95% CI 0.38–0.91, *p* = .017), male sex (HR 0.57, 95% CI 0.37–0.87, *p =* .008*),* primary diabetic kidney disease (HR 0.26, 95% CI 0.12–0.56, *p =* <.001), and time-varying serum albumin levels (HR 0.47, 95% CI 0.35–0.64, *p* < .001) were associated with a reduced risk for peritonitis. In contrast, age (HR 1.02, 95% CI 1.003–1.03, *p =* .015) and time-varying hemoglobin levels (HR 1.1, 95% CI 1.003–1.21, *p =* .044) were associated with an increased risk for peritonitis, as shown in Table 5.

Clinical outcomes in study patients are summarized in Figure 1. Among study patients, the rate of those who remained on PD was 83.1% (69/83) in the NRPR group compared with 82.7% (110/133) in the RPR group. A long-term follow-up in study cohort was until September 2022. The detailed information was shown in Online Resource 5 (Supplemental Table S5).

The comparison results between smokers and nonsmokers were summarized in Online Resource 6–8 (Supplementary Tables S6–S8). Results showed non-significant differences in demographic parameters between smokers and nonsmokers except alcohol drinking and hypertension comorbidity. Smokers demonstrated significantly higher proportion in alcohol drinking and hypertension comorbidity compared to nonsmokers. The comparative results between smokers and nonsmokers in laboratory measurement and PET-related parameters demonstrated that both subgroups had different distribution in blood hemoglobin (Hb), urea nitrogen (BUN), calcium (Ca), sodium (Na), intact parathyroid hormone (iPTH), Kt/*V* (total), and weekly creatinine clearance (Ccr) at different time point, while the alteration of these parameters between both subgroups over time did not show significant difference.

## Discussion

The results of comparison between RPR and NRPR in our study revealed higher percentages of hypertension, coronary artery disease, cerebral stroke, and smoking in the NRPR cohort. This observation implied that physical disability owing to comorbidities in PD patients could interrupt their regular schedule for follow-up in outpatient clinic. To compensate for this inconvenience, a remote monitoring system is likely to promote health management [5,17]. Demographic factors such as age, sex, and marital status were not significantly different between RPR and NRPR. The result was compatible with a previous report [5]. Smokers were more likely to be noncompliant and more likely to skip or shorten treatment in PD regimen [8,18]. Investigators found smokers had lower educational levels and annual incomes [8]. Smokers were also less likely than nonsmokers to describe their global self-health care as good to excellent [8]. In our study, we found a significantly higher proportion of smokers in the NRPR cohort. However, educational levels were not significantly different between the RPR and NRPR cohorts. Nevertheless, smokers’ self-perceived ‘taking care of your health’ values were often lower than those of nonsmokers. In other words, adherence to a PD therapeutic protocol may not be a priority in smokers. It would be useful to further explore the interpretation of the health concept in smokers undergoing PD therapy in a future study. Meanwhile, previous study demonstrated that non-metropolitan PD patients had greater risk for technique failure and shorter time for first peritonitis compared with metropolitan PD patients [19]. In our NRPR cohort, a proportion of PD patients could not adhere to follow-up program per month due to far distance from living residence to PD unit. Consequently, an untoward outcomes of PD therapy in our participants is compatible with previous study.

We examined the changes in laboratory parameters in the present study. In the NRPR cohort, serum albumin showed relatively lower concentrations over two years. Serum calcium and creatinine concentrations showed a significant increase over two years, whereas hemoglobin and serum phosphate concentrations did not present significant changes over two years. Our findings disagreed slightly with those of previous studies. According to a cross-sectional self-report questionnaire study, PD patients with lower adherence rates exhibited higher serum creatinine and phosphate concentrations and lower serum albumin and hemoglobin concentrations compared with those with higher adherence rates [20]. Serum phosphate concentration can be a marker of non-adherence to diet and medication; a similar concept is followed for hemoglobin concentration. In our observation, total Kt/*V* demonstrated a gradual decrease over two years in NRPR subjects. Therefore, inadequate dialysis may not have contributed to the relatively constant serum phosphate concentration over two years in the NRPR subjects. Overall, PD patients with NRPR showed lower concentrations in serum albumin, urea nitrogen, and calcium in our study. This result implied that subjects in the NRPR group were not receiving adequate nourishment. Participants in NRPR group also demonstrated lower serum creatinine levels in 12-month and 24-month period compared to these levels in the participants in RPR group (Table 3). Similarly, weekly total Ccr was lower in participants in NRPR group when compared to those in RPR group (Table 4). These results may indicate lower muscle mass in participants in NRPR group. The relationship between muscle mass and strength with frailty and general practitioner visits have been reported in the research literature [21,22]. We hypothesized that reduced nutritional support and final possible sarcopenia could be one of the reasons to retard physical mobility and regular PD outpatient visits.

In past decades, noncompliant PD patients were commonly reported in skipped or missed PD solution exchanges, which resulted in inadequate dialysis [5,6]. In this study, NRPR patients showed a trend of decreased Kt/*V* values over two years. Moreover, the amount of daily glucose exposure showed an increased trend in this cohort. We also observed that more NRPR patients used automated PD. This is one of possibilities for the discrepancy between these two trends. Another possible reason was the uncontrolled fluid status in patients in the NRPR group and forward to exchange with a higher glucose-based PD solution. Nevertheless, this assumption needs to be validated by further study. However, the PD population in our study in both the NRPR and the RPR groups achieved target adequacy index in PD based on clinical practice guidelines [23]. In other words, non-adherence to regular follow-up in the PD outpatient clinic did not worsen adequate dialysis in our population. Nevertheless, we do not refute the contribution of regular follow-up protocol to PD adequacy.

Noncompliance in PD therapy often increased the incidence of PD-related peritonitis and hospitalization [5,6,20,24]. When examining hospitalization frequency, we found patients with NRPR showed a significantly higher frequency of hospitalization for PD-related causes. However, the frequency of all-cause hospitalizations was not significantly different between the NRPR and RPR cohorts. We also found that RPR may reduce the risk for PD-related peritonitis. Inadequate clearance due to noncompliance to prescribed exchanges is the main assumption for increased risk for PD-related peritonitis and hospitalization in the previous studies [5,6,20]. Patients with NRPR in our study presented with lower PD-adequacy index based on Kt/*V* values and renal creatinine clearance rate. However, we did not measure the missed exchange rate in our patients. Therefore, the definitive causes of inadequate dialysis cannot be ascertained in our study. Similarly, the exact reasons for nearly equal distribution of all-cause hospitalization frequency and drop out percentage between the two cohorts also were obscure in our study.

The present study was subject to several limitations. First, the definition of non-adherence to outpatient follow-upprotocol was arbitrary. Thus, the beneficial effect of a more rigid schedule in an outpatient clinic may be neglected. Further, a cause–effect relationship might not be clearly delineated in our study. Secondly, participants were recruited from two hospitals. Therefore, the influence from hospital-specific effects cannot be eliminated. For example, manpower in individual PD unit, varied routine laboratory measurements, relationship between medical personnel and patients, local cultural characteristics, and reimbursement policy. Thirdly, several aspects of non-adherence to PD therapeutic measures were not examined in our study, e.g., psychological burden, cognitive skills, informative supporting system for patients, and detailed information for PD exchanges. Although staff assumed these circumstances would not associate with non-adherence in PD patients, patients often felt well in the short-run. Therefore, we assumed that these circumstances may not play a greater role in this two-year observational study. Finally, we cannot refute the possibility of protocol deviation in PD home care procedures in our participants. In addition, invasive procedure and infectious diseases could increase the risk of PD-related peritonitis. We reviewed again the assumed causes of PD-related peritonitis in the participants. Majority are related to ignore hygiene procedure in PD solution exchanges. Rare cases were considered the possibility linking to invasive procedures or indolent infection. Accordingly, we suppose that aforementioned events would not influence our final results. A few strengths were identified in the present study. First, this is the first report to examine the influence of PD outcomes using non-adherence outpatient follow-up protocol among incident PD patients. Secondly, our study evaluated the trajectory of laboratory and PD-related parameters over two years. The data provided a real-world scenario of patients after PD initiation. Given the findings, medical staff have gained insight for the necessity of outpatient follow-up in new PD patients. Thirdly, our study demonstrated that smoking and comorbidities associated the motivation to adhere to outpatient follow-up protocols in PD patients. It is not certain if change in lifestyle behavior and social-support provision would enhance adherence. The ultimate challenge will be to develop a suitable intervention to overcome these barriers. Finally, telemedicine has been reported an alternative to ensure compliance in the medical care especially in the COVID pandemic period. The benefit also include reducing the risk for exposure to infectious organisms. Our study may elicit an emerging medicine to apply in the PD patients with high frequency NRPR.

## Conclusion

Non-adherence to regular outpatient follow-up could impact clinical outcomes in new PD patients. Smoking and comorbidities contributed to barriers for adherence to regular outpatient follow-up. Consequently, rates of PD-related peritonitis and hospitalization were higher among patients with non-adherence to regular outpatient follow-up. It is noteworthy that an effective strategy and monitoring procedure for recall adherence is essential for outcome improvement in PD patients.

## Supplementary Material

Supplemental Material
