# Supplementary material for: Is regular in-person recall superior to non-regular in-person recall in clinical outcomes among new patients undergoing peritoneal dialysis
Source: Ren Fail. 2022 Nov 21;44(1):2010–8. doi: 10.1080/0886022X.2022.2145972 (PMC9683045; doi:10.1080/0886022X.2022.2145972)
Supplement: Supplemental Material [file IRNF_A_2145972_SM6691.pdf]

Supplementary Table S1. Standard Operating Procedures(SOPs) for PD recalls

Monitoring parameters	Monitoring interval in Kaohsiung Chang Gung Memorial Hospital in Taiwan (per month)	Monitoring interval in Tianjin First Center Hospital in China (per month)
routine blood test		
Hb	1	1
Alb	1	1
fasting sugar	1	1
BUN	1	1
Cr	1	1
Ca	1	1
P	1	1
Na	1	1
K	1	1
Alkaline phosphatase	1	1
AST	1	1
ALT	1	1
ferritin	3	1
free iron	3	1
TIBC	3	1
cholesterol	3	1
triglyceride	3	1
iPTH	6	1
Kt/V	6	1
Chest x-ray	12	12
Physical examinations		
blood pressure	1	1
body weight	1	
edematous status	1	1
exit site care	1	1
PD home records	1	1
PD nurse: patient (care number)	1:30	1:70
Reimbursement payment (CAPD/APD)($/month)	938/1019	973/973

Abbreviations: Hb: hemoglobin; Alb, albumin; BUN: blood urea nitrogen; Cr, creatinine; Ca: calcium; P: phosphate; Na: sodium; K: potassium; AST: aspartate aminotransferase; ALT: alanine aminotransferase; TIBC: total iron binding capacity; iPTH: intact parathyroid hormone; CXR: chest X ray.

Supplementary Table S2. Comparisons between times to recall and clinical outcomes

Clinical outcome	Times to recall	*P*
Drop-out		
None	2 ± 2.15	1.000
Drop-out	2 ± 1.8	
Peritonitis		
None	2.1 ± 2.18	0.488
Peritonitis	1.74 ± 1.91	
Hospitalization		
None	2.27 ± 2.36	0.389
Hospitalization	1.85 ± 1.96	

Supplement Table S3. PD mode of Incremental PD patients

Variables	(a) Baseline	(b) 12-month	(c) 24-month	Paired *P*		
				a vs b	*a vs c*	*b vs c*
PD mode				0.125	0.006	0.125
CAPD	3 (9.4%)	8 (25%)	13 (41.9%)			
APD	29 (90.6%)	24 (75%)	18 (58.1%)			

Abbreviations: PD, peritoneal dialysis; CAPD, continuous ambulatory peritoneal dialysis; APD, automated peritoneal dialysis.

Supplementary Table S4. PET characteristics

Variables	Total	Non-regular recall	Regular recall	*P*
Baseline				
PD mode				<0.001
CAPD	91 (42.1%)	16 (19.3%)	75 (56.4%)	
APD	125 (57.9%)	67 (80.7%)	58 (43.6%)	
PET				0.214
Low	24 (11.1%)	12 (14.5%)	12 (9.0%)	
Low average	92 (42.6%)	37 (44.6%)	55 (41.4%)	
High average	73 (33.8%)	28 (33.7%)	45 (33.8%)	
High	27 (12.5%)	6 (7.2%)	21 (15.8%)	
Glucose exposure (gm/Day)	112.5 (90-152)	90 (90-120)	130 (97.5-187.5)	<0.001
Urine amount (L/Day)	0.9 ± 0.6	1.0 ± 0.6	0.9 ± 0.6	0.047
Kt/V(total)	2.1 ± 0.5	2 ± 0.5	2.1 ± 0.5	0.195
Total Ccr (weekly) (L/w/1.73m^2)	63.8 (53.8-83.6)	60.2 (49.3-71.3)	65.3 (56.2-87.5)	0.008
Renal Ccr(weekly) (L/w/1.73m^2)	7.2 (3.8-25.8)	32.9 (19.3-46.8)	4.3 (3.2-5.9)	<0.001
12-month				
PD mode				<0.001
CAPD	101 (46.8%)	25 (30.1%)	76 (57.1%)	
APD	112 (51.9%)	56 (67.5%)	56 (42.1%)	
PET				0.005
Low	15 (6.9%)	4 (4.8%)	11 (8.3%)	
Low average	82 (38.0%)	19 (22.9%)	63 (47.4%)	
High average	89 (41.2%)	43 (51.8%)	46 (34.6%)	
High	21 (9.7%)	9 (10.8%)	12 (9.0%)	
Glucose exposure (gm/Day)	121.3 (90-163)	120 (90-160)	130.5 (97.5-187.5)	0.015
Urine amount (L/Day)	0.7 ± 0.6	0.8 ± 0.6	0.7 ± 0.6	0.192
Kt/V(total)	2.0 ± 0.5	1.9 ± 0.5	2.1 ± 0.4	0.001
Total Ccr (weekly) (L/w/1.73m^2)	58.7 (51.2-70.6)	53.1 (45.3-64.3)	61.7 (53.9-73.6)	0.059
Renal Ccr(weekly) (L/w/1.73m^2)	5.1 (3.4-16.2)	18.9 (6.8-33.7)	4.3 (3.2-5.9)	<0.001
24-month				
PD mode				0.003
CAPD	97 (44.9%)	27 (32.5%)	70 (52.6%)	
APD	99 (45.8%)	48 (57.8%)	51 (38.3%)	
PET				0.208
Low	17 (7.9%)	4 (4.8%)	13 (9.8%)	
Low average	79 (36.6%)	25 (30.1%)	54 (40.6%)	
High average	69 (31.9%)	31 (37.3%)	38 (28.6%)	
High	28 (13.0%)	12 (14.5%)	16 (12.0%)	
Glucose exposure (gm/Day)	140 (97.5-181)	140 (110-180)	130.5 (97.5-187.5)	0.864
Urine amount (L/Day)	0.5 ± 0.5	0.5 ± 0.5	0.4 ± 0.5	0.388
Kt/V(total)	2.0 ± 0.4	1.8 ± 0.5	2.0 ± 0.4	<0.001
Total Ccr (weekly) (L/w/1.73m^2)	56.1 (47.8-65.7)	50.6 (40.6-62)	58 (52.3-67.7)	0.232

Renal Ccr(weekly) (L/w/1.73m^2)	4.7 (2.7-9.5)	8.4 (0-19.6)	4.1 (2.9-5.7)	<0.001

Abbreviations: PD, peritoneal dialysis; CAPD, continuous ambulatory peritoneal dialysis; APD, automated peritoneal dialysis; Ccr: creatinine clearance.

Supplementary Table S5. Outcomes in study participants until 9/30/2022

Variables	Total	Non-regular recall	Regular recall	*P*
Patients	216	83	133	
Outcomes				
still on PD(n,%)	54(25%)	19(22.9%)	35(26.3%)	0.686
shift to HD(n,%)	51(23.6%)	10(12%)	41(30.8%)	0.003
Transplant(n,%)	26(12%)	12(14.5%)	14(10.5%)	0.516
Death(n,%)	72(33.3%)	33(39.8%)	39(29.3%)	0.152
Transfer out(n,%)	13(6%)	9(10.8%)	4(3%)	0.035

P-value is estimated using chi-squared test or fisher’s exact test.

Supplemental Table S6. Demography at baseline and clinical outcomes according to smoking status in study population.

Variables	Non-smoker	Smoker	*P*
Patients	188	28	
Cohort			<0.001
Non-regular recall	64 (34.0%)	19 (67.9%)	
Regular recall	124 (66.0%)	9 (32.1%)	
Age (y)	53.3 ± 14.0	47.3 ± 13.2	0.033
BMI(kg/m^2)	23.6 ± 4.0	23.2 ± 3.6	0.578
Sex			<0.001
Male	80 (42.6%)	27 (96.4%)	
Female	108 (57.4%)	1 (3.6%)	
Marital status			0.577
Married	161 (85.6%)	23 (82.1%)	
Others	27 (14.4%)	5 (17.9%)	
Education			0.678
Bachelor	40 (21.3%)	5 (17.9%)	
Others	148 (78.7%)	23 (82.1%)	
Employment status			0.951
Unemployed	142 (75.5%)	21 (75.0%)	
Employed	46 (24.5%)	7 (25.0%)	
Caregiver			0.056
No	152 (80.9%)	27 (96.4%)	
Yes	36 (19.1%)	1 (3.6%)	
Alcohol drinking	9 (4.8%)	11 (39.3%)	<0.001
Etiology of CKD			0.813
Non-DM	137 (72.9%)	21 (75.0%)	
DM	51 (27.1%)	7 (25.0%)	
Comorbidities			
Hypertension	132 (70.2%)	25 (89.3%)	0.035
CAD	58 (30.9%)	11 (39.3%)	0.372
DM	50 (26.6%)	6 (21.4%)	0.561
Cerebral stroke	10 (5.3%)	3 (10.7%)	0.384
Hepatitis B/C	4 (2.1%)	0 (0.0%)	1.000
Clinical outcomes			
Drop-out(n,%)	28 (14.9%)	3 (10.7%)	0.774
Peritonitis(n,%)	38 (20.2%)	7 (25.0%)	0.561
Peritonitis rate (per patient-year)	0.11	0.14	0.264

Peritonitis frequency	0.3 ± 0.7	0.3 ± 0.5	0.952
Hospitalization	111 (59.0%)	20 (71.4%)	0.211
Hospitalization frequency (all causes)	1.5 ± 1.8	1.5 ± 1.4	0.790
Hospitalization frequency (PD causes)	0.6 ± 0.9	0.7 ± 0.8	0.681

P-value is estimated using independent two-sampled t-test, chi-squared test or fisher's exact test.

Supplementary Table S7. Laboratory measurements at baseline and over two-year period.

Variables	Baseline	12-month	24-month
Smoker			
Hb (g/dL)	11.8 ± 1.5	11.9 ± 1.4	11.5 ± 1.6
Alb (g/dL)	3.7 ± 0.6	3.9 ± 0.5	3.8 ± 0.5
BUN (mg/dL)	53.0 (42.0-64.5)	56.6 (48.6-64.6)	57.5 (47.4-64.9)
Cr (mg/dL)	9.5 ± 3.1	10.6 ± 3.2	11.9 ± 3.8
Ca (mg/dL)	8.8 ± 0.8	9.2 ± 1.0	8.8 ± 0.7
P (mg/dL)	4.8 ± 1.5	5.0 ± 1.3	5.6 ± 1.8
Na (mEq/L)	139.9 ± 3.7	141.1 ± 4.2	140.5 ± 4.6
K (mEq/L)	4.4 ± 1.0	4.3 ± 0.7	4.3 ± 0.9
iPTH (pg/ml)	193.5 (99.6-292.9)	244.7 (88.4-302.6)	195.1 (111.6-384.7)
Glucose exposure (gm/Day)	131.2 ± 66.0	143.0 ± 67.0	158.0 ± 69.9
Urine amount (L/Day)	0.9 ± 0.5	0.8 ± 0.7	0.5 ± 0.5
Kt/V (total)	2.0 ± 0.5	1.8 ± 0.5	1.7 ± 0.4
Renal Ccr(weekly) (L/w/1.73m^2)	30.8 (9.4-61.4)	16.5 (3.8-35.6)	9.8 (2.6-20.7)
Total Ccr (weekly) (L/w/1.73m^2)	70.4 (59.6-87.5)	56.7 (51.1-69.8)	55.8 (49.0-62.7)
Non-smoker			
Hb (g/dL)	10.7 ± 1.7	10.5 ± 1.6	10.4 ± 1.4
Alb (g/dL)	3.7 ± 0.4	3.8 ± 0.4	3.7 ± 0.4
BUN (mg/dL)	61.0 (47.8-71.0)	62.0 (52.0-74.0)	57.0 (48.9-71.3)
Cr (mg/dL)	9.7 ± 3.1	10.9 ± 2.9	11.1 ± 3.0
Ca (mg/dL)	8.9 ± 1.1	9.2 ± 0.9	9.2 ± 0.8
P (mg/dL)	5.1 ± 1.2	5.4 ± 1.3	5.5 ± 1.4
Na (mEq/L)	138.9 ± 4.8	138.0 ± 5.0	137.3 ± 5.7
K (mEq/L)	4.2 ± 0.8	4.2 ± 0.7	4.1 ± 0.7
iPTH (pg/ml)	214.4 (103.5-397.0)	256.8 (115.4-378.8)	281.4 (120.3-436.4)
Glucose exposure (gm/Day)	128.4 ± 53.9	136.4 ± 55.9	143.1 ± 58.1
Urine amount (L/Day)	0.9 ± 0.6	0.7 ± 0.6	0.5 ± 0.5
Kt/V (total)	2.1 ± 0.5	2.0 ± 0.4	2.0 ± 0.4
Renal Ccr(weekly) (L/w/1.73m^2)	6.0 (3.7-20.3)	5.0 (3.3-12.6)	4.5 (2.7-8.0)
Total Ccr (weekly) (L/w/1.73m^2)	63.1 (53.5-81.5)	59.3 (51.4-71.1)	56.5 (47.8-65.7)

Abbreviations: Hb: hemoglobin; Alb, albumin; BUN: blood urea nitrogen; Cr, creatinine; Ca: calcium; P: phosphate; Na: sodium; K: potassium; iPTH: intact parathyroid hormone; Ccr: creatinine clearance.

Supplementary Table S8. Comparative results of laboratory measurements and PET-related parameters in study population over time according to their smoking habits.

Variables	NS [a] (over time)	S [a] (over time)	NS vs S [b] (Baseline)	NS vs S [b] (12-month)	NS vs S [b] (24-month)	NS vs S [c] (over time)
Lab measurements						
Hb (g/dL)	0.259	0.661	0.001	<0.001	0.002	0.805
Alb (g/dL)	0.074	0.196	0.995	0.089	0.284	0.373
BUN (mg/dL)	0.264	0.835	0.058	0.006	0.276	0.745
Cr (mg/dL)	<0.001	0.027	0.681	0.632	0.265	0.317
Ca (mg/dL)	0.003	0.096	0.413	0.733	0.015	0.241
P (mg/dL)	0.002	0.171	0.453	0.092	0.898	0.421
Na (mEq/L)	0.019	0.590	0.177	0.001	0.002	0.265
K (mEq/L)	0.374	0.865	0.438	0.576	0.517	0.944
iPTH (pg/ml)	0.302	0.328	0.018	0.429	0.549	0.770
PET parameters						
Glucose exposure (gm/Day)	0.046	0.337	0.829	0.619	0.293	0.759
Urine amount (L/Day)	<0.001	0.029	0.987	0.685	0.722	0.948
Kt/V (total)	0.048	0.076	0.238	0.054	0.001	0.519
Renal Ccr(weekly) (L/w/1.73m^2)	1.000	1.000	0.001	0.058	0.073	1.000
Total Ccr (weekly) (L/w/1.73m^2)	<0.001	0.213	0.555	0.475	0.885	0.772

NS: non-smoker; S: smoker

[a] *P*-value were estimated using one-way repeated measures ANOVA to determine the changes in laboratory measurements and PET-related parameters over time within groups.

[b] *P*-value were estimated using independent two-sample t-test to determine the changes in laboratory measurements and PET-related parameters at single time point between groups.

[c] *P*-value were estimated using two-way repeated measures ANOVA to determine the changes in laboratory measurements and PET-related parameters between groups over times.
